# Evaluation of Etiology and Treatment Methods for Epistaxis: A Review at a Tertiary Care Hospital in Central Nepal

**DOI:** 10.1155/2015/283854

**Published:** 2015-08-09

**Authors:** Ramesh Parajuli

**Affiliations:** Department of Otorhinolaryngology, Chitwan Medical College Teaching Hospital, P.O. Box 42, Chitwan, Nepal

## Abstract

*Introduction*. Epistaxis is one of the most common emergencies in Otorhinolaryngology. It is usually managed with simple conservative measures but occasionally it is a life threatening condition. Identification of the cause is important, as it reflects the management plan being followed. *Aims and Objectives*. To analyze the etiology and treatment methods for patients with epistaxis. *Methods*. A retrospective study was done in a tertiary care hospital in central Nepal. The study period was from May 2014 to April 2015. *Results*. A total of 84 patients had epistaxis; 52 were males and 32 were females. The most common cause of epistaxis was idiopathic (38.09%) followed by hypertension (27.38%), trauma (15.47%), and coagulopathy (8.33%). Regarding treatment methods, most (52.38%) of our patients required anterior nasal packing. Chemical cautery was sufficient to stop bleeding in 14.28% of patients while electrocautery and posterior nasal packing were performed in 2.38% and 16.66% patients, respectively. Two (2.38%) patients required endoscopic sphenopalatine arterial ligation. *Conclusion*. Hypertension, trauma and coagulopathy were the most common etiological factors among the patients in whom etiology was found although in most of the patients etiology could not be found. Anterior nasal packing was the most common treatment method applied to these patients.

## 1. Introduction

Epistaxis is defined as the bleeding from inside the nose or nasal cavity. It is one of the most common emergencies in Otorhinolaryngology worldwide which often requires admission to the hospital [1]. Its incidence is difficult to assess but it is expected that approximately 60% of the population will be affected by epistaxis at some point in their lifetime, with 6% requiring medical attention [2]. Epistaxis can be classified as anterior and posterior epistaxis based on the site of origin [3]. Anterior epistaxis is more common than posterior epistaxis [4]. It usually arises either from kiesselbach's plexus, a rich vascular anastomotic area formed by end arteries, or from vein (retrocolumellar vein). As the bleeding site is accessible, anterior epistaxis which occurs more frequently in children and young adults is rarely serious. On the other hand posterior epistaxis arises from the area supplied by sphenopalatine artery (SPA) in the posterior part of nasal cavity, which is more frequent in elderly people. Usually there is profuse bleeding with difficulty in accessing the site of bleed so it poses challenge in the management. Anterior epistaxis is usually controlled by local pressure or anterior nasal packing while posterior epistaxis often requires posterior nasal packing or arterial ligation.

Epistaxis can be due to both systemic and local factors. Local causes include inflammatory, infective, traumatic, anatomical (deviated nasal septum, septal spur), chemical, or climatic changes, neoplasm, and foreign body. Similarly, the systemic causes of epistaxis are hematological diseases causing coagulopathy, cardiovascular diseases such as hypertension and vascular heart disease, liver disease, renal disease, and anticoagulant drugs. However in majority (80–90%) of patients no identifiable cause is found and is labeled as “idiopathic” [5]. Nose blowing habit, excessive coughing in chronic obstructive pulmonary disease (COPD), straining in constipation and benign prostatic hyperplasia (BPH), and lifting heavy objects are aggravating factors for the epistaxis.

Management of patient with epistaxis at any age group begins with resuscitating the patient, establishing the site of bleed, stopping the bleeding, and treatment of the underlying cause. There is no definite protocol for the management of epistaxis, although various treatment methods are available for the management ranging from local pressure, topical vasoconstrictor, nasal packing, cauterization (chemical/electric), to embolisation or ligation of vessels [6].

The aim of this study is to analyze the patients with epistaxis in terms of etiological factors and treatment methods who required hospitalization.

## 2. Materials and Methods

A retrospective study was carried out among the admitted patients with epistaxis who were managed in the Department of Otorhinolaryngology at Chitwan Medical College Teaching Hospital from May 2014 to April 2015. These patients were received from emergency room (ER), from outpatient department (OPD), or as a referral from other departments. Patients of all ages were included.

All the patients underwent routine investigations such as complete blood count, haemoglobin level, platelet count, random blood sugar, serum electrolytes, urea, creatinine, urine routine examination, and blood grouping. Coagulation profile such as prothrombin time, activated plasma thromboplastin time, and bleeding and clotting time was also performed. Computed tomography (CT) was done in selected cases to rule to neoplasms of nose and paranasal sinuses; and the nasopharynx. Additional investigations were ordered based on history and clinical examination about the possible etiology and comorbidity. Blood sample was also sent for crossmatch when indicated. Beside this, other investigations such as chest X-ray, electrocardiogram (ECG), and serological tests had to be performed for the fitness of procedures requiring general anesthesia, that is, conventional posterior nasal packing and surgical methods to control epistaxis.

Intravenous line was established in all patients with wide bore cannula. Management of the patient began with investigations and treatment side by side. Initially the patients were evaluated with anterior rhinoscopy to identify the site of bleeding. Patients who were brought to ER with complaint of recurrent episodes of excessive bleeding, on whom there was no active bleeding on arrival to the hospital and anterior rhinoscopy did not reveal bleeder, underwent nasal endoscopic examination to search the site of bleeding which might have been located more posteriorly. Treatment of the patients with epistaxis included conservative or nonsurgical treatment and surgical or interventional treatment. Nonsurgical treatment methods included application of topical vasoconstrictors such as oxymetazoline and xylometazoline nasal drop, chemical and electric cauterization of the bleeder, and anterior and posterior nasal packing. Surgical treatment methods were the endoscopic electrocauterization of the bleeder and the endoscopic SPA ligation. All the patients were initially treated conservatively and surgical treatment was considered only when conservative method failed to control the epistaxis. If the bleeder was accessible on anterior rhinoscopy then the patients were treated either with chemical cauterization with silver nitrate with concentration of 75% or with bipolar electrocautery depending on the surgeon's preference. When the bleeder was found to be located more posteriorly on nasal endoscopic examination bipolar electrocautery was used to seal the vessel. If there was diffuse bleeding or when the bleeder could not be located then the patients used to receive anterior nasal packing. Patients with bleeding disorders were packed with absorbable gelatin sponge (Abgel); the rest of the patients received conventional anterior nasal packing with ribbon gauze. Posterior nasal packing was considered in the case of rebleed in a patient who had anterior nasal pack in situ. Surgical methods were the last resort to control bleeding in patients who had recurrent bleed or whose bleeding could not be controlled with those noninterventional methods.

Medical records of those patients were collected and evaluated for the demographics, cause of epistaxis, anatomical location of bleeding site, and the treatment methods provided. Analysis of data was done using SPSS computer software version 16.

## 3. Results

During the study period 84 patients with epistaxis were admitted to this hospital with age ranging from 5 to 86 years. Out of these patients 52 were males and 32 were females. Among these patients 60 (71.42%) presented through ER, 18 (21.42%) presented in OPD, and 6 (7.14%) were received from other departments. According to the type of epistaxis based on site of origin 54 (64.28%) patients had anterior epistaxis and 30 (35.71%) patients had posterior type of epistaxis.

Regarding the etiology, exact cause of epistaxis could not be ascertained in 32 (38.09%) patients, that is, idiopathic. Next common cause was hypertension (23; 27.38%) followed by trauma (13; 15.47%) and coagulopathy (7; 8.33%) (Table 1).

Regarding treatment modalities, conservative/nonsurgical method was sufficient to control epistaxis in most (79; 94.04%) of our patients (Table 2). Among the conservative methods, observation alone without active intervention was carried out in 7 (8.33%) patients. However, 44 (52.38%) patients were treated with anterior nasal packing. Chemical cautery was performed in 12 (14.28%) patients and electrocautery in 2 (2.38%) patients and 14 (16.66%) patients underwent posterior nasal packing. Surgical measures to control epistaxis were carried out in 5 (5.95%) patients. Among these patients 2 (2.38%) underwent resection of tumor; another 2 patients (2.38%) required endoscopic cauterization of SPA while 1 (1.19%) patient required septoplasty to control the epistaxis. Blood transfusion was required in 5 (5.95%) of our patients. None of our patients died due to epistaxis during the study period.

Anterior nasal pack was kept in situ for 48 hours while posterior nasal pack was removed after 72 hours. Broad spectrum antibiotic was used in patients with nasal packing to prevent infectious complications. Similarly, these patients also received antihistaminic and analgesic while the nasal pack was in situ. Antibiotic was also prescribed to the patients who underwent cauterization (chemical or electric) and surgical treatments. Besides these the patients on posterior nasal pack received mild sedation with oral alprazolam to relieve anxiety and pain. All the patients underwent nasal endoscopic examination before their discharge from hospital. Patients were advised to avoid the habits of nose picking and nose blowing if present, to prevent recurrent epistaxis. Patients were discharged from the hospital on oral antibiotic, topical antiseptic cream, and nasal decongestants and were advised to follow up after 1 week.

## 4. Discussion

Patient presenting with epistaxis is frequently encountered in our daily practices. It is common in people of all ages. According to the site epistaxis may be divided into anterior and posterior. Anterior epistaxis occurs more frequently in children and young adults. It is rarely serious as the bleeding point is anteriorly located and is easily identified. Its origin is usually arterial (kiesselbach's plexus) or occasionally venous (retrocolumellar vein). Posterior epistaxis occurs predominantly in the elderly and the site of bleeding is difficult to access as the site of origin is located more posteriorly so it poses a great challenge to arrest bleeding. Age related and cardiovascular diseases related angiopathy changes are probably responsible for the prolonged duration of bleeding. In our study, the age range of the patients varied from 5 to 86 years. Epistaxis was found to be more common in children younger than 10 years (18; 21.42%) and elderly people above 60 years of age (24; 28.57%) which is similar to the results of Pallin et al. [5]. Males were affected more often than females with a ratio of 1.6. Similar findings have been noted in other studies [7, 8]. This may be because the males are more frequently involved in outdoor activities such as sports and interpersonal violence. The higher prevalence of epistaxis in younger children is probably due to their habit of nose picking which causes injury to the kiesselbach's plexus in the anteroinferior part of the nasal septum, that results into anterior epistaxis. Similarly the elderly people commonly have comorbidities such as hypertension and diabetes mellitus which cause degenerative changes in blood vessels making them more fragile which bleed easily on abrupt pressure changes such as straining during micturition and defecation in BPH and constipation respectively; excessive coughing in COPD; and lifting heavy objects. Rhinosinusitis, nasal allergy, temperature changes, and dry heat produce hyperemic nasal mucosa which can bleed while blowing nose or picking nose or with trivial trauma leading to anterior epistaxis [9].

Patient presenting with epistaxis should be thoroughly examined and history should be properly taken to identify the site and cause of bleeding. Most of our patients (32; 38.09%) with epistaxis did not have an identifiable cause which is similar to the study by Christensen et al. [10]. Hypertension was the second most common cause of the epistaxis in our patients which is similar to study by Varshney and Saxena [11]. Nowadays it is said that hypertension is not the cause of epistaxis but it prolongs the bleeding once it starts because in patients with hypertension there is arterial muscle degeneration that leads to defective muscle layer lacking the power to contract resulting in persistence rather than initiation of bleeding. However, the causative factor that might be responsible for the rupture of vessel is still unknown [11]. Some of our hypertensive patients with epistaxis were found to have uncontrolled hypertension due to cessation of antihypertensive medications and inadequate drug therapy because of infrequent check-up; hence the need of regular blood pressure check-up and compliance to the antihypertensive medications should be emphasized. Patients with epistaxis are anxious which might lead to transient hypertension, as the blood pressure was found to be higher in most patients on arrival to hospital. Other causes of epistaxis in our study were trauma (13; 15.47%), coagulopathy (7; 8.33%), infection (4; 4.76%), and tumor (5; 5.95%). The severity of trauma varied from trivial injury such as digital trauma to nasal bone fracture resulting from road traffic accident, physical assault, and sports. Comorbidities found in some of our patients were cardiovascular diseases, diabetes mellitus, and liver and renal diseases. Similarly the aggravating factors found to be associated with epistaxis were COPD, BPH, and constipation.

A variety of treatment methods have been used to control epistaxis which range from nose pinching to ligation of vessels. Method of treatment for epistaxis depends on site, severity, and etiology of bleeding. Treatment modalities can be broadly divided into nonsurgical and surgical approaches. The nonsurgical/conservative modalities include digital nasal compression, topical vasoconstrictor, local cauterization (chemical or electric), and nasal packing (anterior or posterior). If the bleeding point is visible the bleeding site may be sealed either with chemical cautery using silver nitrate, chromic acid, or trichloroacetic acid or with electrocautery using bipolar diathermy. We routinely use silver nitrate for chemical cautery at our institution. Similarly, for electrocauterization bipolar diathermy was used as the monopolar diathermy was associated with risk of optic or oculomotor nerve damage when used in or close to the orbit [12, 13]. Electrocautery can be performed under local anaesthesia in OPD also especially for minor anterior bleed. But for posterior bleed it is better to use electrocautery under general anaesthesia while searching for bleeding point. Nowadays bipolar cautery device with integrated suction tip has also been available. With this single instrument clots can be removed with suctioning which will localize bleeder that can be cauterized easily. Ahmed and Woolford reported 89% success rate with endoscopic electrocautery in patients with epistaxis [14]. If bleeding is not controlled by digital compression and cauterization then anterior nasal packing is done. Anterior nasal packing can be done with nasal tampons such as Merocel and rapid rhino, ribbon gauze, bismuth iodoform paraffin paste impregnated pack (BIPP), or “(absorbable nasal packing materials).” In a study done by Corbridge et al., Merocel nasal packing was found to be effective in 85% of cases, with no difference between the success rates when compared with conventional ribbon gauze [15]. If the bleeding is profuse and not controlled by anterior nasal packing, posterior nasal packing is done. It can be done either by using conventional pack made from gauze piece or Foley's catheter or by using commercially available balloon such as triluminal nasal balloon catheter (Invotec) and Epistat nasal catheter. Conventional posterior nasal packing should be done under general anaesthesia because this procedure is very painful and patient may not tolerate the procedure. Anterior nasal packing is also done once the posterior nasal packing is done. Nonsurgical treatment modalities were effective in most of our patients (79; 94.04%). Among the nonsurgical treatment modalities anterior nasal packing was the most common method followed by chemical cauterization. However, the failure rates of nasal packing in 33 to 40% compared to 3% failure rate of surgical treatment methods have been reported in literature [16]. We used medicated ribbon gauze in most of the patients and nasal tampon “Merocel” in few patients for anterior nasal packing. In our patients with coagulopathy “Abgel” was used to control the bleeding as there was diffuse bleeding from nasal mucosa. Posterior nasal packing was required in 16.66% (14) of our patients after failed anterior nasal packing, which was done either with ribbon gauze or with Foley's catheter followed by anterior nasal packing as well.

Surgical/interventional methods are usually the last resort for refractory epistaxis which does not stop after other means of conservative treatment such as posterior nasal packing. The surgical treatment options include selective arterial embolisation or arterial ligation. Angiographic embolisation uses coils, gel foam, or polyvinyl alcohol to embolise the bleeding vessel. This technique is found to have a success rate as high as 87% [17]. However, arterial embolisation has risk of complications such as cerebrovascular accident, hemiplegia, ophthalmoplegia, facial nerve palsy, and soft tissue necrosis [18]. None of our patients underwent embolisation of vessel. Various surgical techniques exist for ligation of vessels, that is, anterior/posterior ethmoidal artery ligation, internal maxillary artery, or external carotid artery ligation. Ligation of ethmoidal artery can be performed via the external ethmoidectomy incision. Transantral ligation of internal maxillary artery via the Caldwell-Luc approach was a common method to control epistaxis in the past. It was found to be effective in 87% of cases, which is similar to angiographic embolisation [19]. But it is rarely performed nowadays as this procedure is associated with various complications such as sinusitis, facial pain/swelling, oroantral fistula, and paresthesia [20]. Ligation of the external carotid artery has been historically described which is a nonspecific method of decreasing blood flow to the nose which also has frequent treatment failures thought to be due to collateral circulation from opposite external carotid artery [21]. Generally the ligation of external carotid artery is considered a last resort in uncontrolled bleeding when other interventional methods fail. Nowadays the surgical treatment method which has gained popularity among the rhinologists is endoscopic SPA ligation (with clip and electrocauterization or both), which is thought to be more ideal surgical treatment method, as it ligates a major arterial supply and therefore minimizes the risk of refractory epistaxis from collateral circulation. Success rate of 92% to 100% has been achieved with endoscopic SPA ligation [22]. This is a simple and effective method to control refractory epistaxis which also prevents the morbidity and complications of nasal packing. This technique is especially useful in systemically ill individuals who tolerate nasal packing poorly [23]. Failure of this technique is attributed to the failure to identify all the branches of sphenopalatine artery. Endoscopic SPA ligation with bipolar electrocauterization was required in 2.38% (2) of our patients.

There are other newer treatment methods for controlling bleeding such as fibrin glue, which is developed from human plasma cryoprecipitate that binds to the damaged vessels and arrests the bleeding. Randomized controlled trial has found that the local complications due to fibrin glue were lower than that of electrocautery, chemical cautery, and nasal packing. The rebleed rate of fibrin glue was 15% which is comparable to electrocautery [24]. Laser has also been introduced in the management of epistaxis that is found to be useful in cases of recurrent bleeds due to vascular abnormalities such as hereditary haemorrhagic telangiectasia [25].

## 5. Conclusion

Epistaxis is a common emergency condition in Otorhinolaryngology. People of all ages can be affected. Hypertension, trauma, and coagulopathy were the most common etiological/risk factors among the patients in whom etiology was found although in most of the patients etiology could not be found. Conservative or nonsurgical methods were effective to arrest epistaxis in most of the patients. Proper nasal packing is the effective method of controlling epistaxis. Surgical or interventional treatment is only required when epistaxis could not be controlled after nonsurgical treatment methods.

## Figures and Tables

**Table 1:** Etiology of epistaxis (*n* = 84).

Causes	Number of patients	Percentage
Idiopathic cause	32	38.09%
Hypertension	23	27.38%
Trauma	13	15.47%
Coagulopathy	7	8.33%
Tumor (benign/malignant)	5	5.95%
Infection	4	4.76%

**Table 2:** Treatment methods for patients with epistaxis (*n* = 84).

Types of treatment	Number of patients	Percentage
Nonsurgical/noninterventional treatment	79	94.04%
Observation with topical vasoconstrictor only	7	8.33%
Chemical cauterization	12	14.28%
Electrocauterization	2	2.38%
Anterior nasal packing	44	52.38%
Posterior nasal packing	14	16.66%
Surgical/interventional treatment	5	5.95%
Endoscopic SPA ligation	2	2.38%
Excision of bleeding mass	2	2.38%
Septoplasty	1	1.19%
